# Nuclear-encoded factors involved in post-transcriptional processing and modification of mitochondrial tRNAs in human disease

**DOI:** 10.3389/fgene.2015.00079

**Published:** 2015-03-10

**Authors:** Christopher A. Powell, Thomas J. Nicholls, Michal Minczuk

**Affiliations:** Mitochondrial Genetics, Mitochondrial Biology Unit, Medical Research Council, Cambridge, UK

**Keywords:** mitochondria, tRNA, RNA modification, RNA processing, post-transcriptional regulation, mitochondrial disease

## Abstract

The human mitochondrial genome (mtDNA) encodes 22 tRNAs (mt-tRNAs) that are necessary for the intraorganellar translation of the 13 mtDNA-encoded subunits of the mitochondrial respiratory chain complexes. Maturation of mt-tRNAs involves 5′ and 3′ nucleolytic excision from precursor RNAs, as well as extensive post-transcriptional modifications. Recent data suggest that over 7% of all mt-tRNA residues in mammals undergo post-transcriptional modification, with over 30 different modified mt-tRNA positions so far described. These processing and modification steps are necessary for proper mt-tRNA function, and are performed by dedicated, nuclear-encoded enzymes. Recent growing evidence suggests that mutations in these nuclear genes (nDNA), leading to incorrect maturation of mt-tRNAs, are a cause of human mitochondrial disease. Furthermore, mtDNA mutations in mt-tRNA genes, which may also affect mt-tRNA function, processing, and modification, are also frequently associated with human disease. In theory, all pathogenic mt-tRNA variants should be expected to affect only a single process, which is mitochondrial translation, albeit to various extents. However, the clinical manifestations of mitochondrial disorders linked to mutations in mt-tRNAs are extremely heterogeneous, ranging from defects of a single tissue to complex multisystem disorders. This review focuses on the current knowledge of nDNA coding for proteins involved in mt-tRNA maturation that have been linked to human mitochondrial pathologies. We further discuss the possibility that tissue specific regulation of mt-tRNA modifying enzymes could play an important role in the clinical heterogeneity observed for mitochondrial diseases caused by mutations in mt-tRNA genes.

## Introduction

Mitochondria operate a dedicated molecular apparatus to maintain and express their genome. Mitochondrial DNA (mtDNA) encodes 13 vital subunits of the oxidative phosphorylation (OXPHOS) system, the ultimate stage of aerobic cellular energy production. Expression of mtDNA is therefore essential for proper cellular function. Recent research has identified defects in mtDNA expression that are associated with an ever growing and diverse group of human disorders characterized by impaired mitochondrial respiration (Nicholls et al., 2013; Boczonadi and Horvath, 2014). Follow-up studies of patients with respiratory chain disorders, in combination with basic research approaches, has led to the identification of many novel regulatory factors and pathways involved in mitochondrial gene expression. Nonetheless, establishing how defects in these processes contributes to human disease still constitutes a major challenge (Vafai and Mootha, 2012).

The human mitochondrial transcriptome consists of the following key components: two ribosomal RNAs (mt-rRNAs) that are a part of the mitochondrial ribosome, a set of 22 transfer RNAs (mitochondrially encoded tRNAs, mt-tRNAs) and 11 messenger RNAs (mt-mRNAs) coding for the thirteen aforementioned subunits of the OXPHOS complexes (Rorbach and Minczuk, 2012). All of the numerous proteins necessary for mitochondrial RNA synthesis, endonucleolytic processing, post-transcriptional modifications, aminoacylation, stability regulation, and turnover as well as biogenesis of the mitochondrial ribosome and translation within the organelle are encoded by nuclear genes (nDNA) and imported to mitochondria upon translation in the cytosol. Based on published data, evidence from studies of human mitochondrial proteomes (Smith et al., 2012) and our unpublished predictions, we estimate that 250–300 nuclear-encoded proteins are dedicated to serve mitochondrial gene expression.

Numerous genetic defects can lead to perturbations of the OXPHOS system and result in multi-system, often fatal, human diseases. These mutations can be located within nDNA or mtDNA. Isolated OXPHOS deficiencies, that affect a specific biochemical activity of the OXPHOS system, are usually caused by mutations in structural genes (coding for a specific component of the OXPHOS machinery) or in genes encoding proteins responsible for the assembly of a particular respiratory complex. In contrast, inherited pathological mutations, affecting either mitochondrially or nuclearly encoded components of the mitochondrial gene expression machinery, are generally associated with combined OXPHOS deficiencies affecting multiple enzymes involved in cellular respiration. Many mutations in the mtDNA-encoded RNA components of the mitochondrial translation apparatus (mt-rRNAs and mt-tRNAs) have been identified, and detection of yet uncharacterised mutations is relatively straightforward. In contrast, a class of mutations in nuclear gene products involved in proper maintenance of the mitochondrial transcriptome that are linked to human disease has emerged only in recent years. This group currently contains approximately 40 genes (Nicholls et al., 2013; Boczonadi and Horvath, 2014).

**FIGURE 1:**
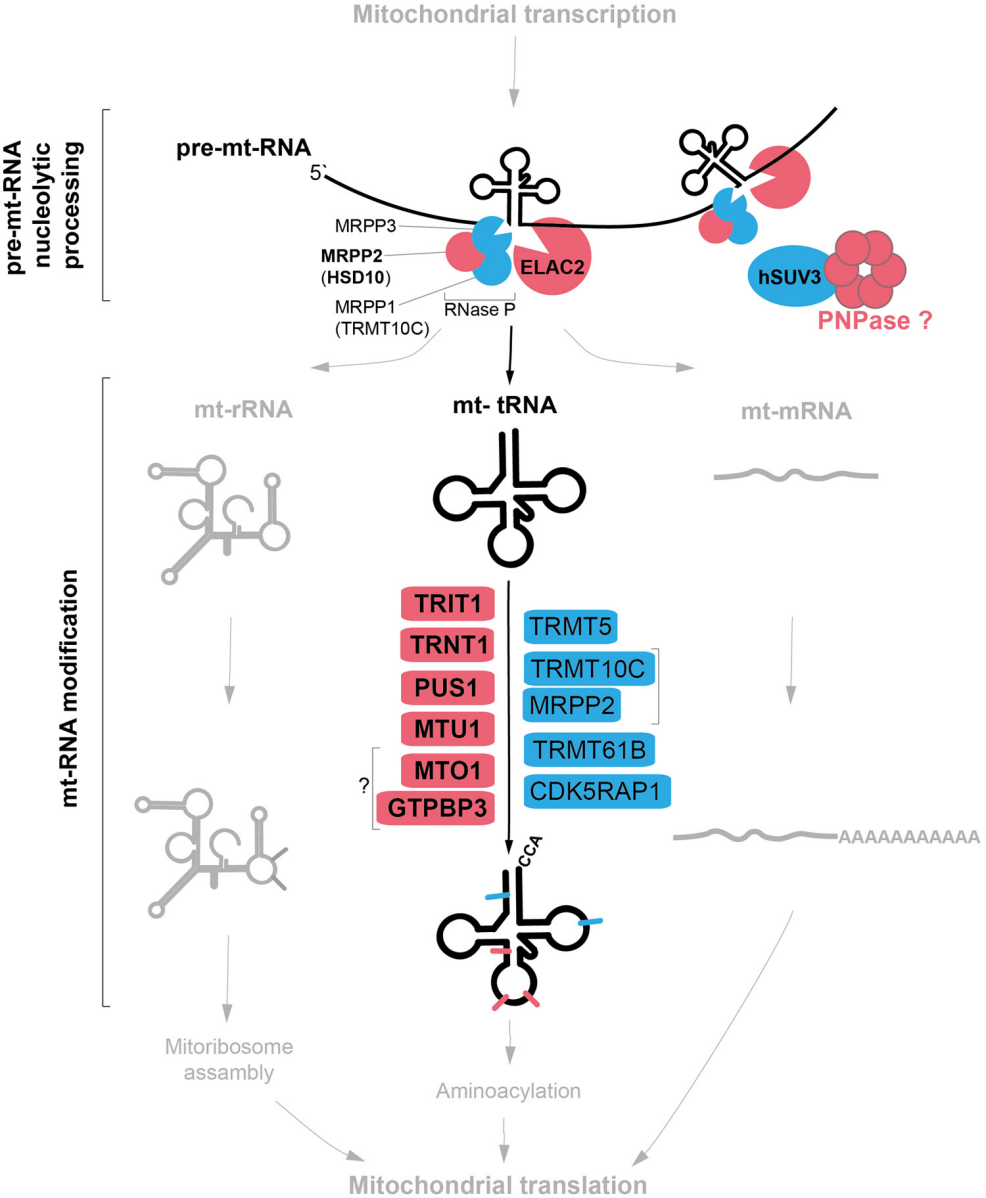
Mitochondrial tRNA maturation and factors involved. Key protein factors involved in post-transcriptional nucleolytic processing and in chemical modifications of mitochondrial tRNAs. Factors associated with human disease are indicated in red; other factors characterized thus far are in blue. Note: PNPase can be either directly or indirectly involved in mt-tRNA processing (red question mark). The bacterial homologs of MTO1 and GTPBP3 form a complex, however, the interaction between the human proteins has not been shown (question mark).

This special-issue review focuses on a subset of nuclear-encoded factors involved in mitochondrial gene expression, for which genetic variants impacting upon mitochondrial pathophysiology have been reported. In particular, we survey the known proteins involved in post-transcriptional nucleolytic processing and nucleotide modifications of mitochondrial tRNAs (**Figure 1**). Key primary pathological mtDNA mutations with recognized effects upon mt-tRNA nucleolytic processing and modifications are also briefly described. Although we recognize the recent discoveries of the key role of mitochondrial aminoacyl-tRNA synthetases (mtARSs) in human pathology affecting mitochondrial translation, this group of disease genes is not covered by the review. Existing recent papers extensively describe the mitochondrial pathologies caused by genetic defects in ARSs (Konovalova and Tyynismaa, 2013; Diodato et al., 2014; Vanlander et al., 2014), including a comprehensive evaluation of the tissue-specificity aspect of ARS mutations presented in this special issue (Euro et al., 2015).

## Processing of mt-tRNA

The two strands of mtDNA are almost entirely transcribed as single polycistronic units from the heavy- and light-strand promoters, HSP and LSP. Transcription from the heavy strand results in a large precursor that encodes 2 rRNAs, 14 tRNAs, and 12 proteins, whereas the light strand codes for eight tRNAs and one mRNA. In these long precursor transcripts, most mRNA and rRNA genes are flanked by tRNAs, whose cloverleaf structures act as recognition elements for endonucleolytic cleavage in order to release individual mtRNA molecules (Anderson et al., 1981; Ojala et al., 1981). Removal of 5′ RNA leaders is carried out by mitochondrial RNase P (Holzmann et al., 2008), while 3′ leaders are removed by an RNase Z activity, which is contributed by ELAC2 (Brzezniak et al., 2011; **Figure 1**). Four mtRNA junctions are not flanked by tRNA genes, and thus do not conform to this paradigm. RNase P has been suggested to be responsible for cleavage of the 5′ end of the CO1 gene (despite the absence of its typical recognition element; Lopez Sanchez et al., 2011), and additional factors are likely required for processing of the remaining junctions.

### 5′-End Processing of mt-tRNA: Mitochondrial Protein Only RNase P

The majority of RNases P discovered thus far are ribozymes, containing a catalytic RNA molecule. Mammalian mitochondrial RNase P, in sharp contrast, is a three-protein complex lacking RNA, apparently assembled from proteins appropriated from other biochemical pathways during evolution to function in tRNA cleavage. These three proteins are: MRPP1 (also known as TRMT10C or RG9MTD1), which is one of three vertebrate homologs of the yeast tRNA-methyltransferase Trm10; MRPP2 (also known as HSD10 or SDR5C1), a protein of the short-chain dehydrogenase/reductase family also involved in isoleucine metabolism; and MRPP3, a metallonuclease-like protein putatively containing the active site of the RNase P complex (Holzmann et al., 2008). MRPP1 and MRPP2 together form a stable subcomplex that also participates in tRNA base modification (discussed in a later section), while the more labile association of MRPP3 completes the RNase P complex (Vilardo et al., 2012). Mutations in MRPP2, encoded by the *HSD17B10* gene on the X-chromosome, are associated with a childhood neurodegenerative disorder, with a number of mutations having been described with varying severity and age of onset (Zschocke et al., 2000; Ofman et al., 2003). A recent study has observed uncleaved precursor mtRNAs in fibroblasts from patients with MRPP2 mutations, as well as in siRNA-treated cultured cells (Deutschmann et al., 2014), consistent with the loss of RNase P activity contributing to the disease progression.

### 3′-End Processig of mt-tRNA: *ELAC2*

Mammals possess two orthologs of the prototypical bacterial RNase Z elaC; a short form (ELAC1) and a long form (ELAC2), both of which are capable of endonucleolytically processing tRNAs *in vitro* (Takaku et al., 2003). ELAC1 predominantly localizes to the cytosol, whereas ELAC2 is targeted both to mitochondria and to the nucleus (Rossmanith, 2011). siRNA downregulation of ELAC2 expression results in an accumulation of 3′ unprocessed mt-tRNA precursors, confirming that ELAC2 is the mitochondrial RNase Z (Brzezniak et al., 2011; Lopez Sanchez et al., 2011). ELAC2 has also been found to associate with the pentatricopeptide repeat (PPR) domain-containing protein PTCD2, which makes a poorly understood contribution to mt-tRNA processing (Lopez Sanchez et al., 2011). Mutations in ELAC2 have been associated with mitochondrial disease in five individuals suffering from an infantile-onset hypertrophic cardiomyopathy and complex I deficiency (Haack et al., 2013). Analysis of patient muscle and fibroblasts revealed a mtRNA processing defect and strongly impaired translation, linking mtRNA processing with the mitochondrial translation machinery (Haack et al., 2013). This highlights the importance of maintaining a correct balance between precursor and mature mtRNAs for proper gene expression in mitochondria, and these aspects require further investigation. In addition to being implicated in mitochondrial disease, ELAC2 is also an established susceptibility gene for prostate cancer (Tavtigian et al., 2001).

### PNPase: A Special Case

A third mitochondrial disease locus with possible links to RNA processing is polynucleotide phosphorylase, PNPase. A proportion of the cellular PNPase pool localizes to the mitochondrial matrix, where it interacts with the RNA helicase SUV3 and participates in mtRNA degradation, possibly of antisense mtRNA molecules (Minczuk et al., 2002; Piwowarski et al., 2003; Nagaike et al., 2005; Borowski et al., 2010, 2013; Szczesny et al., 2010; Chujo et al., 2012). The majority of cellular PNPase, however, localizes to the mitochondrial intermembrane space, spatially separating PNPase from its mtRNA substrates (Chen et al., 2006). This pool of PNPase has a highly unusual proposed role in the import of a small number of cytosolic RNA molecules into the mitochondrial matrix, namely RNase P RNA (the RNA component of the nuclear RNase P ribozyme, suggested to constitute an additional mitochondrial RNase P activity), MRP RNA (hypothesized to be involved in primer formation for mtDNA replication) and 5S cytosolic ribosomal RNA (Wang et al., 2010). The consolidation of these observations with other available data is problematic, however, as (i) the amounts of detectable nuclear RNase P RNA within mitochondria (Puranam and Attardi, 2001) have been suggested to be too low to be functionally relevant (Rossmanith, 2012), while the protein components of the nuclear RNase P complex have also not been found in mitochondria in proteomic studies (e.g., Pagliarini et al., 2008) (ii) MRP RNA has been found to be localized almost exclusively in the nucleolus, with negligible quantities within mitochondria (Kiss and Filipowicz, 1992), and (iii) recent structures of the large mitoribosomal subunit have confirmed that cytosolic 5S rRNA is not a component of the mitoribosome, its role having been replaced by a mt-tRNA (Brown et al., 2014; Greber et al., 2014).

Recessive mutations in the *PNPT1* gene, encoding PNPase, were recently linked to human mitochondrial disease in two siblings affected by encephalomyopathy, choreoathetosis, and combined OXPHOS deficiency, and in three siblings from a second family affected by non-syndromic hearing loss (Vedrenne et al., 2012; von Ameln et al., 2012). In both reports the mutations interfered with the normal oligomerization of PNPase, and the PNPase-mediated import of cytosolic RNAs into mitochondria was found to be impaired. The authors suggested that perturbed import of RNase P RNA impairs the activity of the putative mitochondrially localized nuclear RNase P ribozyme, and thereby inhibit mt-tRNA processing. The effect of these pathological mutations upon matrix mtRNA processing would provide an interesting basis for future study.

## Post-Transcriptional Modifications of Mitochondrial tRNA

**FIGURE 2:**
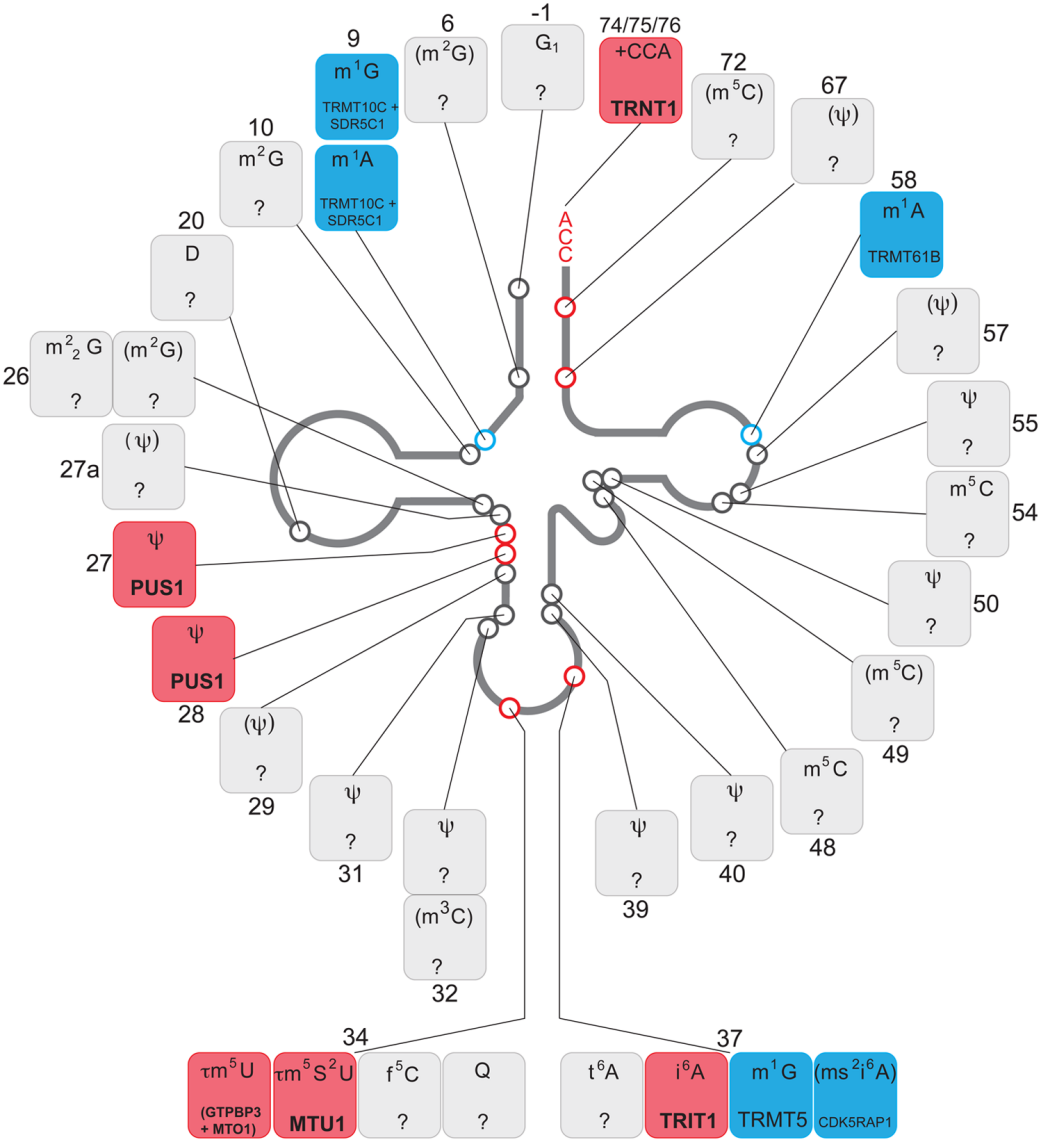
Chemical modifications of mitochondrial tRNA. Schematics of the “clover leaf" secondary structure of a generic mitochondrial tRNA indicating post-transcriptionally modified bases (circles). The details of the chemical modification and enzyme responsible (if known) for each tRNA position is given in boxes, indicating a tRNA base position number next to each box. The chemical modifications identified in mammalian species other than human are in brackets. Color coding: red, enzyme responsible for the modification has been associated with human disease; blue, enzyme responsible for particular modification has been identified; gray, modifying enzyme has not been identified.

The 22 mt-tRNAs act as key adaptor molecules between the mRNAs transcribed from mtDNA and the 13 subunits of the OXPHOS complexes, which they encode. As with all known tRNAs, they undergo numerous post-transcriptional nucleotide modifications prior to becoming active elements in protein translation in mitochondria (Suzuki et al., 2011). Chemical modifications are crucial for tRNA structure and stability and also ensure the efficiency and stringent accuracy that is required during decoding in mitochondrial translation. Mitochondrial tRNA modifying enzymes represent an expanding group of mitochondrial disease causing genes, which we survey below and summarized in **Figure 2** and **Table 1**. We also briefly describe other identified mt-tRNA modifiers (**Table 1**).

**Table 1:**
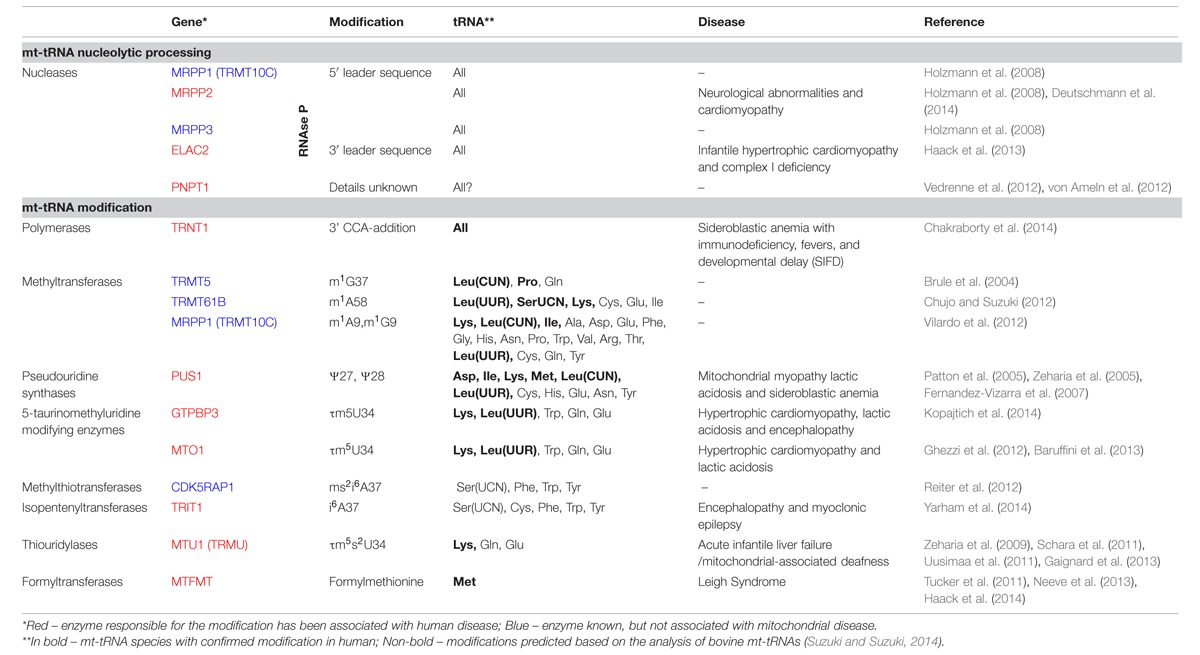
Mitochondrial tRNA processing and modification in human disease.

### Polymerases

Almost all eukaryotic tRNA genes, along with many archaeal and bacterial tRNA genes, encode an RNA molecule that lacks crucial nucleotides from the fully matured tRNA following cleavage from the precursor RNA. Further polymerization is subsequently required to produce the full length tRNA, with the same process often utilized for the repair of damaged tRNA ends.

### TRNT1

The universally conserved CCA sequence found on the 3′ terminus of all tRNAs represents the major post-transcriptional polymerization event involved in the maturation process. Once matured, the newly polymerized 3′ terminus acts as the amino acid attachment site, catalyzed by the cognate aminoacyl-tRNA synthetase. Studies on *in vitro* transcribed tRNAs have reported that the CCA sequence acts as a tRNase Z anti-determinant, ensuring that a futile cycle between polymerase and ribonuclease activities does not occur (Mohan et al., 1999). Additionally, the CCA sequence existing in the form of a tandem CCACCA on the 3′ end has been identified as a quality control signal, targeting misfolded or hypomodified tRNAs for degradation (Wilusz et al., 2011). In human mitochondria, polymerization at the 3′ terminus of tRNAs is performed by the essential enzyme, TRNT1 (tRNA-nucleotidyltransferase 1), in a non-templated reaction (Nagaike et al., 2001). Despite the lack of any template to specify the sequence with which nucleotides need to be incorporated, TRNT1 exhibits extremely high selectivity for its CTP and ATP substrates, in the correct order, with precise termination following the completion of the CCA sequence. Primary mitochondrial disorders as a consequence of impaired CCA-addition have been known for some time, resulting from mutations affecting either the catalytic rate (m.4317A > G in tRNA^Ile^; Tomari et al., 2003) or the substrate binding strength (m.3303C > T in tRNA^Leu(UUR)^; Levinger et al., 2004). Recently, a cohort of patients exhibiting SIFD (Sideroblastic anemia associated with immunodeficiency, periodic fevers and developmental delay) were reported to carry mutations in *TRNT1*, with the range of clinical severity correlating with the degree of TRNT1 loss of function (Chakraborty et al., 2014). The role of mitochondrial dysfunction in congenital sideroblastic anemia has been well demonstrated, with causative mutations found in genes involved in haem synthesis, mitochondrial iron–sulfur biogenesis, and mitochondrial translation (Fleming, 2011). The phenotypic pleiotropy observed in SIFD may be a consequence of the dual localization of TRNT1 resulting in impaired cytosolic as well as mitochondrial translation.

### THG1L

Despite the overwhelming preference among polymerases for 5′-3′ synthesis, as occurs during the CCA-addition described above, a unique case of 3′-5′ polymerization is utilized to catalyze the addition of an uncoded guanine onto the 5′ end of tRNA^His^. This additional 5′-guanylate (G_-1_), a signature characteristic of tRNA^His^ molecules from all but a handful of species, is simply encoded in the genome of bacteria and many archaea, whereas in eukaryotes it is, in most cases, added post-transcriptionally by a member of the tRNA-histidine guanyltransferase 1 (Thg1) family. G_-1_ has been shown to serve as an important discrimination element for its cognate histidine aminoacyl-tRNA synthetase, with depletion of the yeast Thg1 being followed by a concomitant diminished aminoacylation rate. In humans, the mitochondrial tRNA^His^ gene does not encode for an additional 5′-guanine, and a member of the Thg1-family has been predicted within the nuclear genome (Nakamura et al., 2013), which may represent a candidate gene for mitochondrial diseases due to its critical role in translation efficiency.

### Methyltransferases

Of the plethora of modifications identified in mitochondrial tRNAs, methylation represents the most abundant and diverse grouping, being found at a wide range of different locations within a tRNA, as well as at different positions within a given base. Depending on the nature of the modification and the location of the residue, methylations within tRNAs have been implicated in structural stabilization, a decrease in the likelihood of frameshifting, and in improving translation efficiency.

### TRMT1

The first identified human tRNA methyltransferase, TRMT1, was found to be responsible for both the m^2^G26 and m^2^_2_G26 tRNA methylations based on the activity of the recombinant protein *in vivo* in *Escherichia coli* cells expressing human cytosolic tRNA^Tyr^ (Liu and Straby, 2000). The predicted mitochondrial targeting sequence, along with the mitochondrial localization of the yeast homolog, Trm1 (Ellis et al., 1989), has lead to the conclusion that TRMT1 is responsible for the m^2^_2_G26 modification found in human mitochondrial tRNAs. A homozygous frameshift mutation predicted to completely inactivate TRMT1 has been reported in a family with non-syndromic intellectual disability (Najmabadi et al., 2011).

### TRMT5

The recombinant protein approach has also been utilized to characterize a second human tRNA methyltransferase, TRMT5, which was found to catalyze the formation of m^1^G37 *in vitro* for a number of tRNA substrates, including human mitochondrial tRNA^Pro^ (Brule et al., 2004). In both yeast and bacteria, the m^1^G37 modification has been shown to play a critical role in preventing frameshifting errors at the ribosome (Urbonavicius et al., 2001).

### TRMT61B

The *N*^1^-methylation of adenosine at position 58, which confers stabilization to the tertiary structure of the tRNA (Robertus et al., 1974), has been identified in three human mitochondrial tRNAs: Leu(UUR), Lys, and Ser(UCN). Through a combination of *in vitro* and *in vivo* analysis, m^1^A58 has been recognized as the product of a mitochondria-specific methyltransferase, TRMT61B (Chujo and Suzuki, 2012).

### TRMT10C (MRPP1)

As mentioned previously, a subcomplex of RNase P performs a tRNA modifying role entirely separate from the endonucleolytic cleavage of the complex as a whole. The *N*^1^-methylation of purines at position 9 (R9) is carried out by a complex of MRPP1 (also known as TRMT10C), a homolog of yeast tRNA:m1R9 methyltransferase Trm10, and MRPP2 (also known as SDR5C1 or HSD10), a dehydrogenase whose exact role in the R9 methylation complex remains unknown (Vilardo et al., 2012). Methylation of position 9 has been demonstrated to play a critical role in tRNA structure, being both necessary (Sakurai et al., 2005) and sufficient (Helm et al., 1999) for the correct folding of a tRNA. Patients with HSD10 disease, caused by mutations in MRPP2, present with progressive neurological abnormalities and cardiomyopathy and also have depleted levels of TRMT10C. However, the potential involvement of diminished m^1^R9 in mitochondrial tRNAs in the pathomechanism of HSD10 disease has yet to be demonstrated (Deutschmann et al., 2014).

### Pseudouridine Synthetases

The isomerization of uracil produces the most abundant of the modifications so far described; pseudouridine (Ψ, or 5-ribosyluracil), which is bestowed with unique chemical properties largely thanks to an additional hydrogen bond donor on its non-Watson Crick edge which often acts to rigidify an RNA structure (Davis, 1995; Newby and Greenbaum, 2002) and contributes toward the stabilization of a particular structural motif. The* in situ* isomerization of uracil is performed by members of the pseudouridine synthase family, which exhibit remarkably high specificity for their target residues. For this reason, organisms typically utilize a large number of pseudouridine synthase proteins in order to target the wide range of pseudouridylation sites within their substrate RNAs. Of the 13 pseudouridine synthases found in humans, PUS1 is by far the most well characterized, and has been found to be responsible for Ψ27 and Ψ28 in mitochondrial tRNAs (Patton et al., 2005), as well as catalyzing the formation of Ψ in a number of other non-coding RNAs (e.g., Zhao et al., 2004). Loss of function mutations in PUS1, along with a concordant decreased rate of mitochondrial translation, have been identified in a number of patients with myopathy, lactic acidosis and sideroblastic anemia (MLASA; Patton et al., 2005; Zeharia et al., 2005; Fernandez-Vizarra et al., 2007). Studies in yeast and *E. coli* have provided candidate genes for the remaining pseudouridine sites found in human mitochondrial tRNAs, such as PUS6 for Ψ31 (Ansmant et al., 2001), PUS9 for Ψ32 (Behm-Ansmant et al., 2004), TRUB2 for Ψ55 (Becker et al., 1997; Zucchini et al., 2003), and PUS3 for Ψ39 and Ψ40 (Lecointe et al., 1998), however, the enzymatic role of these proteins have yet to be demonstrated in humans. In contrast to the stabilizing effects of pseudouridylation, uridine may also be modified to dihydrouridine (D), allowing for greater conformational flexibility through the saturation of its base (Dalluge, 1996). The human enzyme DUS2 is proposed to introduce D20 into mitochondrial tRNAs based on yeast homology (Xing et al., 2004), and has been implicated in pulmonary carcinogenesis (Kato et al., 2005).

### Taurine Modification Enzymes (MTO1 AND GTPBP3) and Thiouridylase (MTU1 aka TRMU): A Special Case

Non-canonical base pairing at position 34, also known as the wobble base, allows for the expansion of the codon-recognition capabilities of a tRNA and therefore all possible codons to be decoded. As a consequence of this critical function, position 34 is one of the most frequently modified nucleosides, with a wide variety of modification chemistries, in order to specify unusual base pairing features. The modification at position 34 in mammalian mt-tRNAs commonly involves the incorporation of taurine onto an encoded uridine in the form of 5-taurinomethyluridine (τm^5^U) found in mammalian tRNA^Leu(UUR)^, tRNA^Trp^, tRNA^Lys^, tRNA^Gln^, and tRNA^Glu^ (Suzuki and Suzuki, 2014). Interestingly, pathogenic mutations in tRNA^Leu(UUR)^ (3243 and 3271) and tRNA^Lys^ (8344) that are known to cause MELAS and MERRF, respectively, are found to dramatically diminish the level of the U34 modification in the corresponding tRNA (Suzuki et al., 2002). It is postulated that these mutations disrupt the recognition determinants utilized by the enzyme(s) responsible for the synthesis of the modification, with the resulting impairment of codon recognition being the primary cause of disease (Kirino et al., 2005, 2006), however, the definitive identification of the responsible enzymatic activity has yet to be demonstrated in humans. Studies in bacteria and yeast mitochondria have greatly assisted efforts due to the high evolutionary conservation of the enzymes involved, despite the fact that both incorporate a carboxymethylaminomethyl (cmnm) group in place of taurine. In *E. coli*, MnmE and GidA form a heterodimeric complex that catalyzes the formation of cmnm at position 5 of the wobble uridine (Moukadiri et al., 2009), as do the related mitochondrially localized MSS1 and MTO1 in yeast. The human homologs have been identified as GTPBP3 and MTO1 (Li and Guan, 2002; Li et al., 2002), both of which have been localized to mitochondria and are able to complement mitochondrial phenotypes in corresponding yeast deletion strains. More recently, patients exhibiting hypertrophic cardiomyopathy and lactic acidosis as a consequence of a combined respiratory chain deficiency have been found to carry mutations in *MTO1* (Ghezzi et al., 2012; Baruffini et al., 2013) and *GTPBP3* (Kopajtich et al., 2014). An association of *MTO1* mutations with impaired mitochondrial translation remains to be shown for human cells. However, the striking similarity in clinical presentation suggests a common pathomechanism related to the U34 modification for both diseases. Notably, in addition to hypertrophic cardiomyopathy, lactic acidosis, and combined OXPHOS deficiency, some patients with *GTPBP3* mutations also developed encephalopathy (Kopajtich et al., 2014). Through linkage analysis, both MTO1 and GTPBP3 have also been implicated as modifier genes of a homoplasmic mitochondrial deafness-associated m1555A > G mutation within 12S rRNA (Bykhovskaya et al., 2004). m.1555A > G presents with a broad range of phenotypic severity, the variance of which is believed to be influenced by the inheritance of nuclear-encoded modifier genes (Bykhovskaya et al., 1998). Interestingly, a m.1477C > G mutation disrupting the corresponding base pair in yeast 15S rRNA was found to be required in combination with MSS1 and MTO1 mutants in order to cause respiratory deficiency (Decoster et al., 1993; Colby et al., 1998).

Of the five tRNAs previously discussed as substrates of MTO1 and GTPBP3, three of these, tRNA^Lys^, tRNA^Gln^, and tRNA^Glu^, undergo further modification on the wobble base to produce 5-taurinomethyl-2-thiouridine (τm^5^s^2^U) (Suzuki et al., 2002). A thiouridylase, MTU1 (also known as TRMU), catalyzes the formation of the 2-thio group in mitochondrial tRNAs, with its depletion resulting in their dramatically reduced thiolation (Umeda et al., 2005; Sasarman et al., 2011). The formation of 2-thiouridine is also proposed to be dependent on a cysteine desulfurase, NFS1, a component of the Fe/S cluster assembly machinery, for supplying the sulfur atom (Nakai et al., 2004). Mutations in MTU1, as well as those within its substrate tRNA, have been found in a large number of individuals presenting with reversible infantile respiratory chain deficiency, characterized by acute liver failure (Zeharia et al., 2009; Schara et al., 2011; Uusimaa et al., 2011; Gaignard et al., 2013). Limited sulfur availability during the neonatal period has been proposed as the explanation for the spontaneous recovery of patients following a window of vulnerability, and cysteine supplementation during this period may act as a potential treatment (Boczonadi et al., 2013).

### Other tRNA-Modifying Enzymatic Activities

The anticodon loop is a hotspot for post-transcriptional modifications, and harbors the greatest range of modifications within the tRNA, predominantly at positions 34 and 37. Position 34 undergoes a greater range of modifications than those so far described, an observation in line with its critical role in the fine-tuning of codon recognition. The ability of wobble base modifications to drastically alter codon–anticodon interactions is clearly illustrated in the case of 5-formylcytidine (f^5^C34) in mt-tRNA^Met^ (Moriya et al., 1994). In the cytosol, a single codon, AUG, encodes for methionine and is recognized by two different tRNAs, one for initiation and one for elongation (Mayer et al., 2001). In mitochondria, however, methionine encoding is expanded to AUA, as well as AUG, with both codons being recognized by a single tRNA bearing a CAU anticodon (Bilbille et al., 2011). The modification of C34 to f^5^C34 is believed to expand the codon recognition capabilities of tRNA^Met^ through enhanced binding to AUA (Lusic et al., 2008; Takemoto et al., 2009). The enzymatic activity responsible for f^5^C34 formation has yet to be identified. The final modified base thus far found at position 34 of human mitochondrial tRNAs, queuosine (Q), represents an interesting case in which rather than an encoded base being modified *in situ*, the entire base (in this case a guanine) is excised and replaced through a breakage of the glycosidic bond. The substitution of guanine for Q is performed by tRNA-guanine transglycosylases (TGTases), with QTRTD1, one of two mammalian TGTases, believed to perform the reaction in human mitochondria (Boland et al., 2009; Chen et al., 2010). As with previous wobble base modifications, Q34 has been implicated in modifying the decoding capabilities of a tRNA (Morris et al., 1999).

Position 37, a purine in all human mt-tRNAs, is methylated in the case of guanine, as mentioned above. A37 on the other hand is commonly modified through the addition of an isopentenyl group, and the resulting i^6^A37 promotes translational efficiency and fidelity through increased decoding stringency, with its loss resulting in mitochondrial dysfunction in yeast (Lamichhane et al., 2013a). The tRNA isopentenyltransferase responsible for i^6^A37 formation in humans, TRIT1, was initially identified as a tumor suppressor gene (Spinola et al., 2005), and its siRNA-mediated depletion causes diminished i^6^A37 in mitochondrial tRNAs (Lamichhane et al., 2013b). Mutations in TRIT1, with a corresponding loss of the i^6^A modification and compromised mitochondrial translation, have been identified in siblings with encephalopathy and myoclonic epilepsy (Yarham et al., 2014). A subset of TRIT1 substrates are further modified through the action of a mitochondrially localized methylthiotransferase, CDK5RAP1, to form ms^2^i^6^A37 (Reiter et al., 2012). The final modification found at position 37 in human mitochondrial tRNAs, threonylcarbamoyl adenosine (t^6^A), has also been demonstrated to play a critical role in maintaining decoding accuracy (Stuart et al., 2000). Its biosynthetic pathway in humans remains unknown, however, with YRDC and OSGEPL1, the human homologs of the yeast *N*(6)-threonylcarbamoyltransferases Sua5 and Qri7, respectively, being the most likely candidates (Oberto et al., 2009; Wan et al., 2013).

### Methionyl-tRNA Formyltransferase (MTFMT): Special Case

Although not a chemical modification of tRNA *sensu stricto*, it is worth mentioning that mt-tRNA^Met^ is also formylated on the methionine amino acid charged to the 3′ end to form fMet-tRNA^Met^, which increases its affinity toward the mitochondrial initiation factor (IF2_mt_; Takeuchi et al., 1998). Mutations in mitochondrial methionyl-tRNA formyltransferase (MTFMT) have been identified in a number of patients presenting with mitochondrial encephalomyopathy (Tucker et al., 2011; Neeve et al., 2013; Haack et al., 2014).

## Primary mtDNA Mutations Affecting RNA Processing and Post-Transcriptional Modifications

It is important to note that defects in mtRNA processing by nuclear-encoded mitochondrial enzymes may also result from mutations in the RNA substrate itself, if recognition or cleavage of the RNA by the processing machinery is impaired. However, while mutations in the nDNA involved in mtRNA processing, as described above, would be expected to interfere with all mtRNA processing events, primary mtDNA mutations would only be expected the affect the mtRNA bearing the mutation. The multi-copy nature of the mitochondrial genome also means that mutant and wild-type genomes coexist within a cell (heteroplasmy) with clinical phenotypes only manifesting above a threshold level of typically 80–90% of mutated genomes. Pathological mutations in mt-tRNA genes are the most frequent among all mtDNA mutations (Yarham et al., 2010). Given the structural determinants of post-transcriptional mt-tRNA nucleolytic processing and modification, many of these mutations are likely to destabilize tRNA structure and interfere with these processes. Below we present some examples of pathogenic point mutations in mt-tRNA genes that have been linked to a mtRNA processing or modification defect. The m.4308G > A mutation in the mt-tRNA^Ile^ gene has been found to cause chronic progressive external ophthalmoplegia (CPEO). This mutation is believed to destabilize the secondary structure of the tRNA, making the tRNA a poor substrate for 3′ end processing by ELAC2 (Schaller et al., 2011). A number of well-studied mutations in the mt-tRNA^Leu(UUR)^ gene, frequently associated with mitochondrial myopathy, encephalopathy, lactic acidosis, and stroke-like episodes (MELAS), have also been found to interfere with precursor mtRNA processing. Disease-associated point mutations in mt-tRNA^Leu(UUR)^ have been found to impair RNaseP and ELAC2-mediated tRNA cleavage both *in vitro* (Rossmanith and Karwan, 1998) as well as in transmitochondrial cybrids and in patient fibroblasts (Bindoff et al., 1993; Levinger et al., 2004). However, a number of other molecular defects have also been reported for mt-tRNA^Leu(UUR)^ carrying MELAS-associated point mutations, such as decreased aminoacylation (Chomyn et al., 2000), decreased CCA-addition (Levinger et al., 2004), structural defects (Wittenhagen and Kelley, 2002), decreased stability, and loss of post-transcriptional modification of the wobble-base (Yasukawa et al., 2000). It is therefore very difficult to determine how each of these factors contributes to the overall pathophysiology of the mutation, as the observed processing defect may merely be a side-effect of the impaired folding or post-transcriptional processing/modification of the tRNA. This complicated relationship between mt-tRNA point mutations and post-transcriptional events implies that a detailed analysis of mt-tRNA processing and modifications could be helpful in elucidating the pathomechanism of particular mt-tRNA mutations.

## mt-tRNA Metabolism and Tissue Specificity of Disease

**FIGURE 3:**
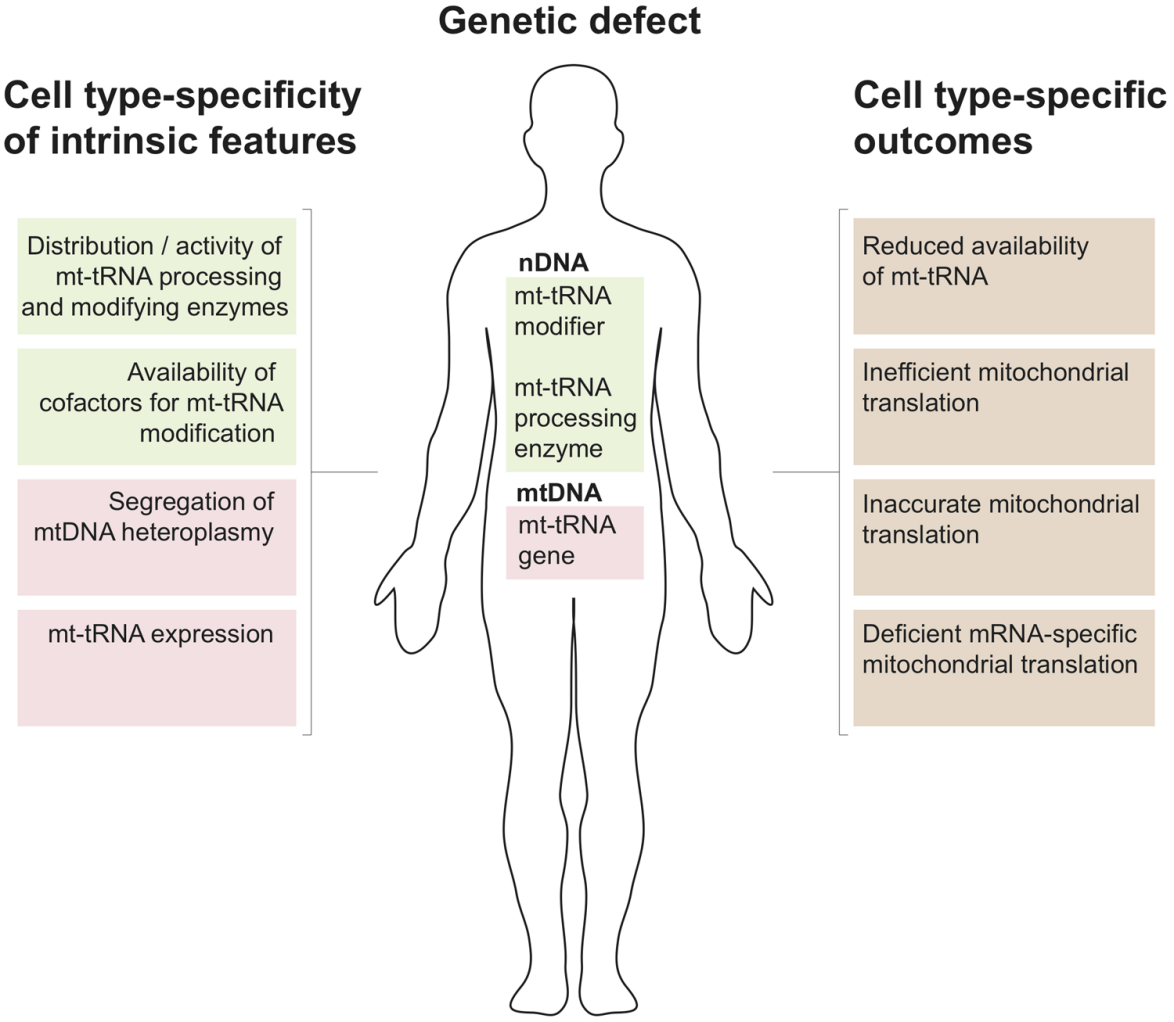
Mitochondrial tRNA metabolism and tissue specificity of associated disease. Several properties that relate to tissue-specific regulation of mitochondrial tRNA biology by nuclearly- (green) and mitochondrially encoded genes (red) are listed to the left. Possible genetic defect leading to perturbations of mitochondrial tRNA metabolism resulting from mutations in the nuclear (nDNA) or mitochondrial genome (mtDNA) are shown in the center. The tissue specific regulation of the mt-tRNA maturation pathways in conjunction with nDNA or mtDNA mutations can give rise to the cell type-specific molecular phenotypes listed to the right.

The above survey shows that pathological mutations in mitochondrial tRNA processing and modifying enzymes are numerous and result in divergent clinical phenotypes. Organ specificity of the observed pathological symptoms is an important feature of these cases (e.g., for mutations in *GTPBP3* and *MTO1* – mostly heart, in *TRIT1* – brain and in *MTU1* – liver). This is unexpected given that any defect in mt-tRNA metabolism should, in principle, perturb only a single cellular process, which is mitochondrial translation. The molecular mechanisms behind this phenomenon are largely unknown. Below we discuss key aspects of mtRNA metabolism and possible outcomes associated with defective mt-tRNA biology that might be helpful when formulating new hypotheses on the different clinical presentations of mt-tRNA-related genetic defects (**Figure 3**)>.

According to recent data, approximately 25 tRNA positions contain modified nucleotides in mammalian mitochondria. Some key tRNA positions, such as 34 and 37 (**Figure 2**), contain different modifications depending upon the mt-tRNA species. Therefore, is has been estimated that over 30 mitochondrial enzymes are required for introducing these modifications (Suzuki and Suzuki, 2014). The tissue-specific distribution and/or activity of these enzymes are expected to be different. Also, cellular distribution may vary between enzymes, with, for example, PUS1 and TRIT1 being localized to both the nucleus and mitochondria (see above). The same may apply to the nucleases responsible for mt-tRNA cleavage from the primary transcript. As a consequence, organ-specific regulation of the level of specific mt-tRNA modifications and the concentration of certain mt-tRNA species is expected to take place. Furthermore, given the chemical intricacy of mt-tRNA modifications (for example τm^5^s^2^U or ms^2^i^6^A, **Figure 2**), biosynthesis of the cofactors to be introduced into the mt-tRNA molecules rely on a number of apparently unrelated biochemical pathways. This links the mt-tRNA modifications to metabolic properties of certain tissues, where the availability of individual substrates might be considerably different, thereby indirectly influencing protein synthesis in mitochondria. Moreover, the molecular genetics of mtDNA maintenance and expression may have a key role in regulating the properties of a mt-tRNA population. The segregation of mtDNA heteroplasmy is known to be tissue-dependent, which is of great importance for the clinical outcomes in the case of the primary mtDNA mutations in mt-tRNA genes. Also, the rate of mtRNA synthesis and decay is different in various human cell types, regulating the specific concentration of mt-tRNAs.

Given the above tissue-specificity of the intrinsic features governing mt-tRNA biology, genetic defects either in the nDNA coding for mt-tRNA processing or modifying enzymes or in mitochondrial tRNA genes can be expected to have cell type-specific consequences. Several molecular scenarios might be envisaged for this to happen (**Figure 2**). For example, chemical modification of tRNA nucleotides are often crucial for stability, with hypomodified tRNAs being targeted for degradation (Alexandrov et al., 2006). Thus a genetic defect in a mt-tRNA modifier responsible for stabilizing mt-tRNA may result in cell type-specific RNA decay leading to local mt-tRNA insufficiency. For example, the binding of protein partners may act to stabilize an otherwise unstable tRNA, as has been demonstrated for the m.3243A > G mutation in mt-tRNA^Leu(UUR)^ (Perli et al., 2014) or the m.1624C > T mutation in mt-tRNA^V al^ (Hornig-Do et al., 2014) following the overexpression of a non-catalytic portion of LARS2. Tissue specificity may then result from the loss of a stabilizing mt-tRNA modification due to differing expression levels of these protein partners among tissue types. Another instance may occur for a pathogenic mutation that partially inactivates a mt-tRNA modifier, combined with a tissue-specific availability of a cofactor used by this enzyme. In such a scenario the pathological outcome of this mutation will be more devastating in cofactor-poor tissues, leading, for example, to defects in mitochondrial translation accuracy and efficiency (as proposed for sulfur availability and MTU1 – see above). Furthermore, tissues with high demands for mt-tRNA expression might be more affected by mutations in mt-tRNA processing or modifying enzymes owing to their reduced activity. In some cases, the lack of modification of certain mt-tRNAs might affect the translation of only a subset of mitochondrial transcripts that are rich in codons recognized by this particular mt-tRNA. In such a situation isolated, rather than combined, OXPHOS deficiency might be produced. Lastly, tissue-specific segregation of pathological mtDNA mutations affecting mt-tRNA has been well documented since the origin of mitochondrial medicine. As mentioned above, these mutations can affect processing or modification of mt-tRNAs. It is conceivable that particular tissue might be severely affected as a compound effect of high mt-tRNA mutation load in conjunction with low activity of enzymes responsible for maturation of this impaired substrate mt-tRNA.

## Concluding Remarks and Future Directions

Recent research has revealed the important role of mitochondrial tRNA post-transcriptional nucleolytic processing and chemical modifications in human disease (**Figures 1** and **2**; **Table 1**). However, the mechanistic details for the origin of pathologies associated with defects in mt-tRNA biology remain poorly understood (**Figure 3**).

It is now time to intensify efforts on elucidating the molecular machinery and mechanisms responsible for regulating post-transcriptional stages of mt-tRNA biology. Given that over 30 different modifications to mammalian mt-tRNAs have been described so far, with only 10 enzymes responsible for their synthesis being currently described (**Figure 2**), special attention should be given to assigning the remaining two-thirds of mt-tRNA modifiers. This point is particularly important for mitochondrial medicine, as despite recent advances in next-generation sequencing technologies, defining the genetic architecture of the many unsolved cases of mitochondrial disease remains a major challenge. Further research should also concentrate on revealing the consequences of the presence of hypomodified or incorrectly processed mt-tRNAs on mitochondrial protein synthesis. To address this, it will be essential to apply novel technologies such as mitoribosome profiling based on next-generation sequencing of RNA (Rooijers et al., 2013) and/or on mass spectrometry (Wessels et al., 2013). It will also be important to analyze which mt-tRNA mutations, the most common class of pathogenic mtDNA mutation, affect post-transcriptional processing and modification. This research will help to understand the remarkable heterogeneity of human pathologies associated with defects of mt-tRNA metabolism. It is also our belief that these efforts will stimulate further studies aimed at the development of novel therapeutics based on modulation of mt-tRNA processing and, in particular, modification, leading to effective treatments for these otherwise untreatable mitochondrial diseases.

## Conflict of Interest Statement

The Editor Daniele Ghezzi declares that, despite having collaborated with author Michal Minczuk, the review process was handled objectively and no conflict of interest exists. The authors declare that the research was conducted in the absence of any commercial or financial relationships that could be construed as a potential conflict of interest.
